# A Quality Improvement Project to Measure and Improve the Quality of Below-Knee Ankle Cast Application in Emergency Departments

**DOI:** 10.7759/cureus.57479

**Published:** 2024-04-02

**Authors:** Matthew W Pritchard, Michael Apostolides, Paul J Baggott, Liam Donnelly

**Affiliations:** 1 Surgery, Royal North Shore Hospital, Sydney, AUS; 2 Orthopaedics and Trauma, William Harvey Hospital, Ashford, GBR; 3 Trauma and Orthopaedics, Royal Surrey County Hospital, Guildford, GBR; 4 Orthopaedics, Chelsea and Westminster Hospital, London, GBR

**Keywords:** clinical audit, orthopaedics & traumatology, plaster cast immobilization, emergency departments, ankle fractures

## Abstract

Background

Ankle fractures are very common injuries seen in an emergency setting. Initial management involves the application of below-knee plaster casts. At our local trauma meetings, we have observed that below-knee casts are often applied incorrectly which can result in suboptimal outcomes for patients and increase the burden on plaster room services if re-application is required. This quality improvement project aimed to assess the quality of below-knee cast applications for ankle fractures in two local district general hospitals (DGHs).

Methodology

We performed a closed-loop audit utilising a retrospective analysis of patients who underwent casting for unstable ankle fractures. Two audit cycles were completed over a 90-day period across two DGHs. Working within our local orthopaedic unit, we created a targeted, multi-disciplinary educational programme led by experienced plaster technicians. Between audit cycles, we organised a single interactive session with specialist nurses in the urgent treatment centre (UTC) of our DGH while a second DGH did the same with junior doctors working in the emergency department. Both sessions demonstrated correct casting techniques and discussed the importance of a neutral ankle position for optimal patient recovery. Our audit criteria were based on AO Foundation guidance, which states that the ankle should be immobilised in a neutral plantigrade position. All patients with an unstable ankle fracture requiring immobilisation in a below-knee cast were included in the audit. We measured the angle of plantarflexion from neutral, with 90° representing a neutral angle. The angle between the axis of the tibia and the sole of the foot was measured and judged to be within an acceptable range if it was between 80° and 100°, representing a stable ankle position. The audit findings were presented in our local audit meeting.

Results

In our first audit cycle, we collected data from 65 patients across both sites (N = 32 for DGH 1 and N = 33 for DGH 2). The mean angle was 108.5° and 18 of the 65 (27.7%) patients had angles of ankle plantarflexion that were in the acceptable range (80°-100°). Following the intervention, we again collected data from 61 patients across both sites (N = 28 for DGH 1 and N = 33 for DGH 2). The mean angle was 106.2° and 23 of the 61 (37.7%) patients had an acceptable angle of ankle plantarflexion (80°-100°). Both of our outcome measures showed an improvement but were not statistically significant. The hospital that provided an educational session for the doctors showed an improvement in acceptable ankle casts of 3% while the hospital which provided an educational session for the UTC team improved by 22%.

Conclusions

We demonstrated a quantifiable approach to assess and improve the quality of below-knee cast application for ankle fractures via a single intervention that would be easily reproducible in other hospitals. We suggest further studies to investigate below-knee cast application quality and its association with patient outcomes as our data and other preliminary sources suggest that current standards are unsatisfactory.

## Introduction

Ankle fractures are one of the most common injuries seen in emergency departments and have increased in prevalence over recent years [1-3]. For displaced ankle fractures, reduction should be performed as soon as possible after initial assessment to reduce pain and swelling and prevent skin necrosis [4-7]. Once reduced, the fracture should be stabilised in a well-fitted below-knee (also known as a short leg) back-slab cast or splint, with the limb elevated and a post-reduction plain radiograph arranged to confirm adequate alignment [4].

The majority of below-knee back-slab casts are applied in the urgent treatment centre (UTC) or emergency department. Depending on fracture stability, patients are either discharged home and followed up in a fracture clinic, or admitted for surgical fixation [8]. The goal of cast application is to achieve stabilisation of the ankle joint. Neutral ankle positioning with the foot at 90° to the leg is critical for maximal joint stability. AO Foundation guidance [9] states that ‘for the ankle, a functional position corresponding to 90° of flexion is advised’ and that ‘it is important to preserve a plantigrade foot position, with the plantar surface parallel to the ground when the tibia is upright.’ Poor casting techniques often causes discomfort for the patient and can lead to circulatory and nerve impairment, compartment syndrome, malunion, instability, skin breakdown, and joint stiffness [8,10]. Moreover, unsatisfactory casting requires plaster technicians to reapply casts, placing an additional burden on already busy plaster room services [8]. Previous reports have demonstrated that below-knee casts for ankle fractures are generally applied incorrectly [8].

The aim of this quality improvement (QI) project is to measure and improve the quality of below-knee casting for ankle fractures at two district general hospitals (DGHs). Specifically, we aimed to assess ankle ‘neutrality.’ This report outlines our project with the hope it may serve as a template for QI in other hospitals.

## Materials and methods

The first audit cycle used below-knee cast radiographs from 32 patients in a DGH (DGH 1) and 33 patients from another (DGH 2), collected retrospectively, over a 90-day period to measure the baseline of ankle neutrality and cast acceptability (defined as 80°-100°, with 90° representing a neutral angle). All patients included in the audit had suffered an unstable ankle fracture which required immobilisation in a below-knee cast. The quality of the below-knee cast application was assessed using lateral view ankle radiographs. The angle between the axis of the tibia and the sole of the foot was measured and judged to be within an acceptable range if it was between 80° and 100°, representing a stable ankle position. All radiographs were taken from the hospital’s picture archiving and communication system (Carestream Vue PACS®) and the angle was measured using integrated software.

The QI project team in our DGH (DGH 1) presented the baseline data at the local orthopaedic audit meeting. We identified that cast application was most commonly performed by UTC specialist nurses and junior doctors working in the emergency department. We assumed the most likely cause for poor application was lack of knowledge and, therefore, arranged a single interactive session led by experienced plaster technicians for the UTC team while a QI project team in the other DGH (DGH 2) arranged a single session for junior doctors. The session focussed on the below-knee cast application technique and highlighted the importance of neutral plantarflexion of the ankle. Additionally, educational posters were placed at hospital sites where plastering occurred, highlighting the importance of achieving a neutral ankle position. The team then re-audited the data after the intervention using a sample of 28 (DGH 1) and 33 (DGH 2) below-knee cast radiographs from their respective hospitals.

Statistical analysis of ankle neutrality was performed using Student’s t-test. Statistical significance for the study was set at p-values <0.05. In addition, the team used Pearson’s chi-squared test to determine if patients were more likely to have an acceptable plantarflexion angle (defined as 80°-100°) post-intervention compared to pre-intervention.

## Results

**Figure 1:**
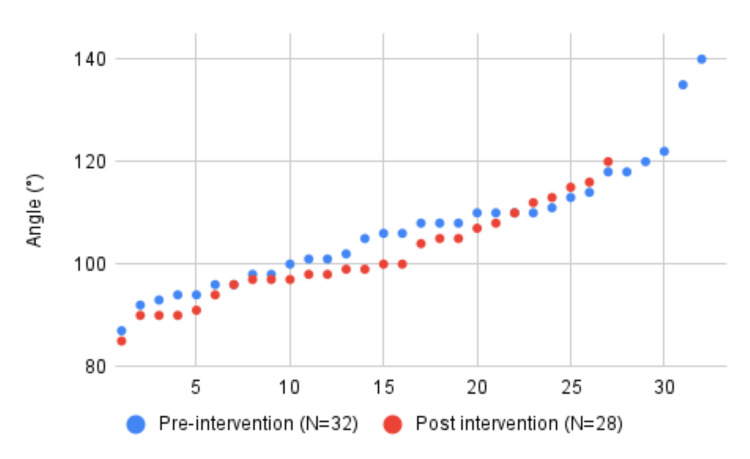
Pre-intervention and post-intervention graph of the angle of plantarflexion. District general hospital 1.

**Figure 2:**
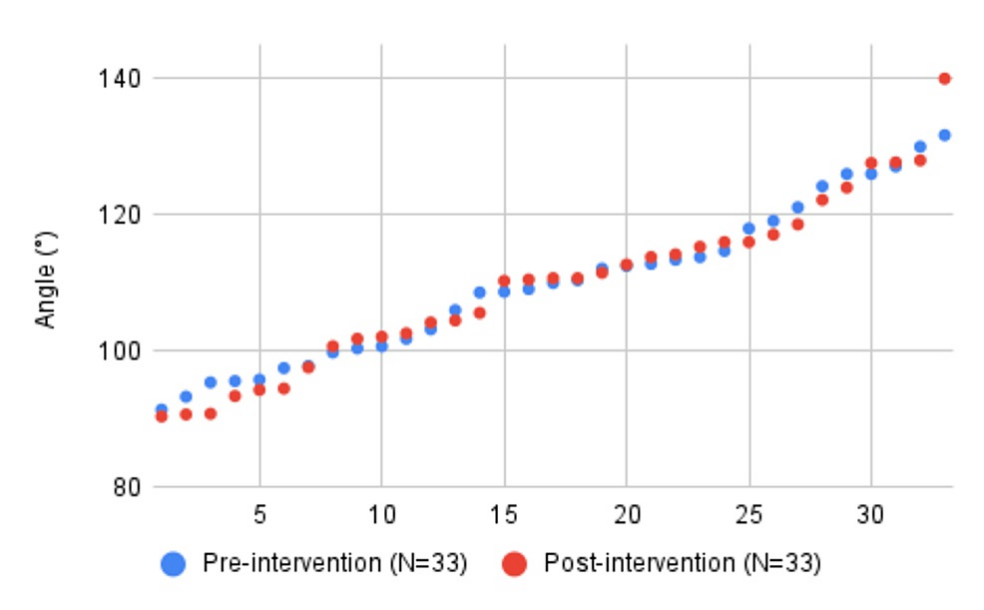
Pre-intervention and post-intervention graph of the angle of plantarflexion. District general hospital 2.

The first audit cycle included 65 (N = 32 for DGH 1 and N = 33 for DGH 2) patients across both sites. The range of angles of ankle plantarflexion was between 88° and 140° with a mean angle of 108.5° (standard deviation (SD) = 11.7, 95% confidence interval (CI) = 105.6°-111.3°). Overall, 18 of the 65 (27.7%) patients had ankle plantarflexion angles of 80°-100° and were acceptably neutral. Following the intervention, we again collected data from 61 patients across both sites (N = 28 for DGH 1 and N = 33 for DGH 2). The range was between 85° and 144° with an average of 106.2° (SD = 11.9, 95% CI = 103.3°-109.2°]. Further, 23 of 61 (37.7%) patients had ankle plantarflexion angles between 80° and 100°. The pre-intervention and post-intervention results for each hospital are shown in (Figures 1, 2).

The combined data showed an overall improvement in the angle of plantarflexion of the ankle after intervention from 108.5° to 106.2° (p = 0.285). The number of acceptable ankle casts in our study after our intervention improved from 18/65 (27.7%) to 23/61 (37.7%) (p = 0.231). Both of our outcome measures showed an improvement but were not statistically significant. The hospital which provided an educational session for the doctors showed an improvement in acceptable ankle casts of 3% while the hospital which provided an educational session for the UTC team improved by 22%.

## Discussion

On review of ankle radiographs during trauma meetings at two DGHs, senior orthopaedic staff noted an unsatisfactory application of below-knee plaster casts for ankle fractures, with concerns regarding ankle neutrality. Our results showed that a significant proportion (72.3%, n = 47/65) of below-knee casts were applied with the ankle in an unsatisfactory position. Evidence states that better ankle positioning yields better patient outcomes and possibly reduces the number of cast reapplications [8,10]. Although this study did not investigate the rate of cast reapplication, a similar UK-based study reported that 20% of below-knee casts required reapplication and inadequate reduction was seen in 31% of cases [8]. We believe that the quality of below-knee cast application for ankle fractures is likely unsatisfactory in many hospitals; however, we are unable to substantiate this viewpoint due to a lack of relevant literature.

The findings of our QI project failed to demonstrate a statistically significant improvement in the quality of below-knee cast application following a single intervention at two DGHs. We suspect that our small sample size caused the results to lack statistical significance. In contrast, a QI project by Williams et al. [8] demonstrated a statistically significant improvement in ankle neutrality after implementing regular practical training sessions and displaying information posters in clinical areas. In addition, Williams et al. [8] showed that fewer below-knee cast reapplications were required following their interventions but their small sample size precluded statistical significance. We were unable to identify any other studies in the literature to consolidate these findings. Interestingly, in our study, the hospital that designed an intervention targeting the UTC team (DGH 1) produced more improvement than the hospital that targeted junior doctors (DGH 2). This could reflect the frequency of cast application by different teams which is likely to vary at each site. Additionally, junior doctors rotate to different specialties while UTC team members are likely to stay in the role, meaning they are more likely to be present in our re-audit.

We do note some limitations to our QI project. First, there are several other parameters to assess the quality of cast application in addition to ankle neutrality, including the degree of fracture reduction, comfort, fit, and durability [8]. Thus, considering ankle neutrality in isolation may not entirely represent cast application quality. Second, measurements of ankle plantarflexion were performed by a single reviewer, reducing reliability and introducing possible bias.

## Conclusions

We demonstrated a quantifiable way to assess and improve the quality of below-knee cast application for ankle fractures via a single intervention that would be easily reproducible in other hospitals. We suggest further studies to investigate below-knee cast application quality and its association with patient outcomes as our data and other preliminary sources suggest that current standards are unsatisfactory.
